# Rebound effect following intravitreal bevacizumab in branch retinal vein occlusion

**DOI:** 10.4103/0974-620X.53042

**Published:** 2009

**Authors:** Satyen Deka, Kruto Kalita, Sunil Kumar Singh

**Affiliations:** Vitreoretina Service, Sri Sankaradeva Nethralaya, Guwahati, India

Bevacizumab (Avastin, Genentech Inc., San Francisco, CA) is a 149 kD humanized monoclonal antibody directed against all isoforms of vascular endothelial growth factor A (VEGF-A), approved by the US Food and Drug Administration (FDA) in February 2004, for intravenous use in metastatic colorectal cancer. Off-label intravitreal injections of bevacizumab (Avastin) have been given for the treatment of various neovascular and exudative ocular diseases since May 2005. [1,2] We report rebound effect following intravitreal bevacizumab injection in branch retinal vein occlusion (BRVO).

A 50-year-old man, presented with a two weeks’ history of blurred vision in his left eye. He gave past history (before five years) of vitreous hemorrhage in his right eye for which vitrectomy was advised, but not done. Past medical history was remarkable for hypertension, dyslipidemia, and eczema. His best-corrected visual acuity (BCVA) in the right eye was hand movement close to the face and, 20/30 N8 in the left eye.

Slit lamp examination of the anterior segment was unremarkable and both eyes had normal intraocular pressure. Four mirror gonioscopy was normal in both eyes with 360 degrees open angle. The right fundus was not visible and examination of left fundus showed superotemporal BRVO with macular edema [Figure 1A]. Fundus fluorescein angiography (FFA) of the left eye [Figure 1B] confirmed the diagnosis of BRVO. Optical coherent tomography showed macular edema [Figure 1C]. After discussing treatment options and obtaining informed consent, an off-label intravitreal injection of bevacizumab 1.25 mg in 0.05 ml was administered in his left eye. After one-month treatment with bevacizumab injections, his left eye vision improved to 20/20 N6 with significant resolution of BRVO and macular edema. The follow-up after two months revealed visual acuity of 20/20 N6 with further resolution of BRVO and macular edema [Figure 2]. One month later he reported again with sudden blurring of vision in the left eye of three days duration. On examination BCVA in his left eye was 20/30 N8 and there was rebound appearance of BRVO [Figure 3A]; FFA corroborated the clinical findings [Figure 3B].

**Figure 1A:**
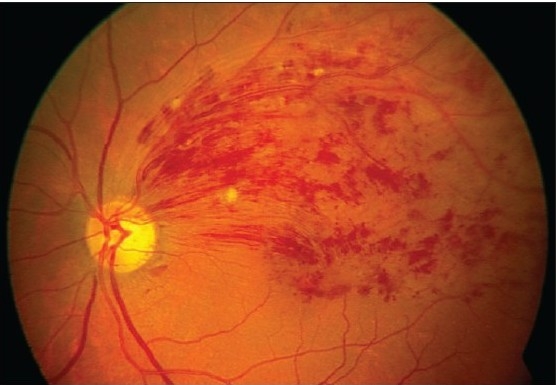
Color fundus photograph at initial presentation showing branch retinal vein occlusion with typical retinal hemorrhage and macular edema

**Figure 1B:**
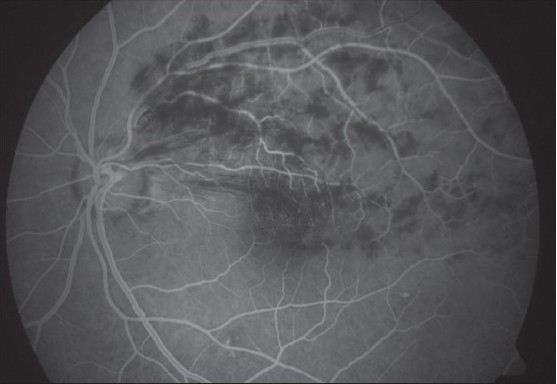
Fundus fluorescein angiogram at initial presentation

**Figure 1C:**
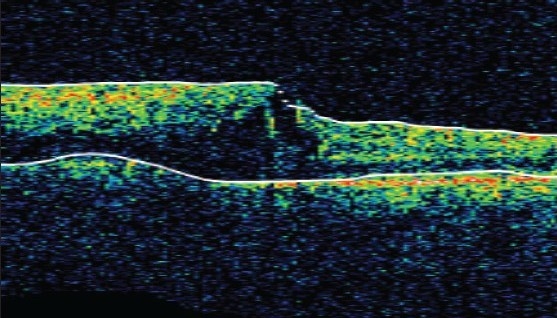
Optical coherent tomography showing macular edema (480 micron)

**Figure 2:**
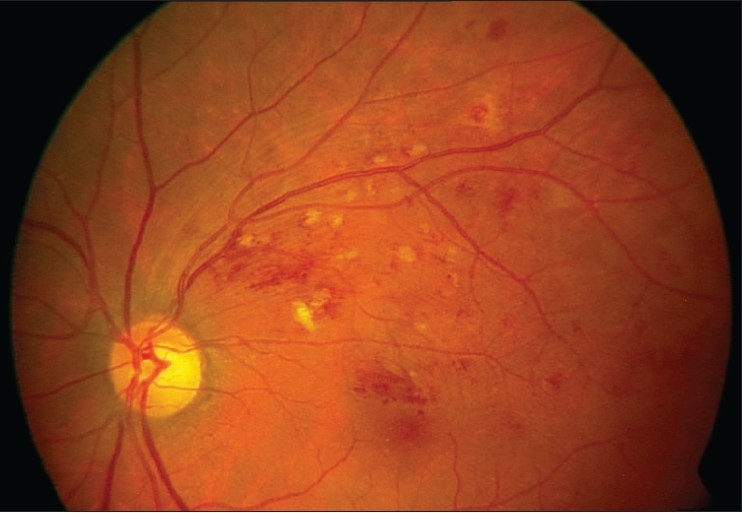
Fundus photograph showing partial resolution of branch retinal vein occlusion and macular edema two months after intravitreal bevacizumab

Laser photocoagulation and systemic steroid has been traditionally used for patients with BRVO. The tissue damage from laser and its disappointing visual restoration led to the development of anti-VEGF therapies. Bevacizumab is an alternate treatment option for BRVO, which offers significant advantages over steroid and laser treatment. It can be instituted without delay unlike laser which necessitates clearing of retinal hemorrhages. Using intravitreal bevacizumab, a positive biologic effect has been observed with significant resolution of BRVO and macular edema. [3,4] Literature suggests that the cumulative probability of developing a second episode of the same or a different type of retinal vein occlusion in the same eye was 0.9% within two years and 2.5% within four years. [5] Our case showed rebound effect following intravitreal bevacizumab injection and a recurrence of BRVO involving the same arteriovenous crossing site after three months. We hypothesize that intravitreal bevacizumab gives temporary benefit in BRVO and attribute the rebound effect to falling drug levels.

**Figure 3A:**
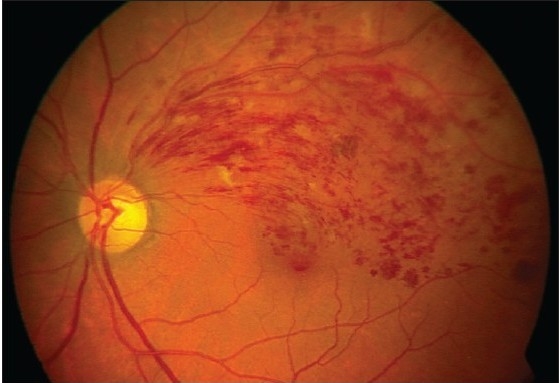
Fundus photograph showing rebound effect three months after intravitreal bevacizumab in branch retinal vein occlusion

**Figure 3B:**
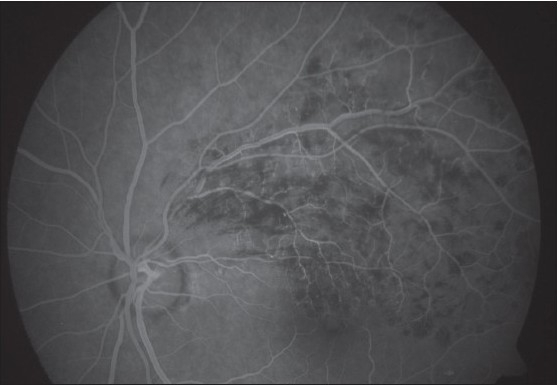
Fundus fluorescein angiogram three months after intravitreal bevacizumab
